# Impaired retrieval processes evident during visual working memory in schizophrenia

**DOI:** 10.1016/j.scog.2016.07.002

**Published:** 2016-08-11

**Authors:** Peter A. Lynn, Seung Suk Kang, Scott R. Sponheim

**Affiliations:** a Minneapolis VA Health Care System, 1 Veterans Drive, Minneapolis, MN, 55417, United States; b University of Minnesota, Minneapolis, MN, 55455, United States

**Keywords:** Working memory, Schizophrenia, Event-related potential, Endophenotype, Relatives

## Abstract

Prominent working memory (WM) deficits have been observed in people with schizophrenia (PSZ) across multiple sensory modalities, including the visuospatial realm. Electrophysiological abnormalities noted during early visual processing as well as later cognitive functions in PSZ may underlie deficiencies in WM ability, though the mechanisms linking behavior to neural responses are not well understood. WM dysfunction has also been observed in biological relatives of PSZ (REL) and therefore may be a manifestation of genetic liability for the disorder. We administered a delayed response visuospatial WM task to 23 PSZ, 30 of their REL, and 37 healthy controls (CTRL) to better understand the contributions of neural abnormalities to WM performance deficits associated with schizophrenia. PSZ performed more poorly on the WM task and failed to effectively process distractor stimuli as well as CTRL and REL. N1 electrophysiological responses to probes during retrieval differentiated the type and locations of stimuli presented during encoding in CTRL. Retrieval N1 responses in PSZ, however, failed to do so, while retrieval responses in REL showed more pronounced differentiation of stimulus features during encoding. Furthermore, neural responses during retrieval predicted behavioral performance in PSZ and REL, but not CTRL. These results suggest that retrieval processes are particularly important to efficient visuospatial WM function in PSZ and REL, and support further investigation of WM retrieval as a potential target for improving overall WM function through clinical intervention.

## 1 Introduction

Working memory (WM) dysfunction in people with schizophrenia (PSZ) has been demonstrated across various sensory modalities (Fleming et al., 1997, Haenschel et al., 2007, Lee and Park, 2005). WM deficits have likewise been observed in the unaffected first-degree relatives of PSZ (Conklin et al., 2000, Park et al., 1995, Pirkola et al., 2005, Seidman et al., 2012), suggesting that WM impairment may represent an endophenotypic marker for schizophrenia (Gottesman and Gould, 2003, Haenschel and Linden, 2011).

In addition to WM performance deficits, related neurophysiological abnormalities have been demonstrated in PSZ and their unaffected relatives. Deficient early visual processes have been repeatedly observed in PSZ during WM tasks (Dias et al., 2011, Haenschel et al., 2007, Zhao et al., 2011), and related deficits have been observed in unaffected relatives who presumably carry genetic liability for the disorder (Yeap et al., 2006). Electrophysiological correlates of later cognition, including WM functions (Haenschel et al., 2007, Zhao et al., 2011), have likewise been shown to be abnormal in PSZ. Some abnormalities in later processes have similarly been reported in unaffected relatives (Lee et al., 2010, Sponheim et al., 2006). Recent work in WM has focused on the role of distracting stimuli in preventing efficient encoding which may compromise the amount or content of material in WM in PSZ (see Eich et al., 2014, Erickson et al., 2014). However, researchers have yet to understand the mechanisms linking these neural abnormalities to observed behavioral deficits during WM in PSZ and their unaffected relatives.

We analyzed event-related potentials (ERPs) elicited during WM encoding and retrieval from PSZ, their unaffected relatives, and nonpsychiatric controls to better understand the contribution of neural responses to WM dysfunction associated with the disorder. To understand neural mechanisms associated with WM performance deficits, we examined electrophysiological responses to task manipulations related to distracting stimuli, amount of material (i.e., load), and the location of the probe stimulus. If, for example, PSZ and/or relatives showed abnormal modulation of neural responses to distractors versus target stimuli, this would support the notion that distracting stimuli may be particularly important in explaining WM deficits in these populations. Similarly, examination of responses during encoding and retrieval would allow for isolation of neural deficits to a particular component of WM. We expected to see ERP abnormalities in PSZ and their first-degree relatives as compared to controls, as have been previously observed in studies outside the realm of WM and only scarcely investigated in visuospatial WM, especially in unaffected relatives. Specifically, we hypothesized that PSZ alone would show increased late potential amplitudes, thought to index WM load, for distractor stimuli during encoding, suggesting that PSZ were encoding task-irrelevant information. In addition, we hypothesized that REL would show stronger abnormalities in neural indices than behavioral indices.

## 2 Methods

### 2.1 Participants

Participants (*n =* 90) were 23 PSZ, 30 first-degree biological relatives of PSZ (REL), and 37 healthy controls (CTRL; Table 1). They were enrolled as part of a family study of severe psychopathology based at the Minneapolis Veterans Affairs Medical Center. PSZ were recruited through a mental health clinic and past research rosters, other current studies of severe psychopathology, referrals from physicians, as well as community-based mental health facilities and the medical center. REL were recruited using contact information provided by PSZ, and CTRL were recruited primarily through advertisement in the medical center and community, as well as from past research rosters. Enrolled participants underwent clinical assessments, the results of which were subjected to a consensus diagnosis process in which two or more Ph.D. clinicians or advanced doctoral students reviewed participants' study materials to form jointly agreed upon diagnoses. Full inclusion and exclusion criteria and clinical assessments are described in the supplementary materials.

**Table 1:** Participant characteristics.

	CTRL (*n* = 37)	PSZ (*n* = 23)	REL (*n* = 30)	Test Statistic (Degrees of Freedom)	*p*-value
% Female	35.1%	4.3%	63.3%	*χ*^2^(2) = 19.6	*p* < .001
Age (years)	46.7 (11.1)	42.9 (10.2)	45.3 (10.7)	*F*(2, 87) = 0.9	*p* = .42
Years of education	15.1^a^ (1.9)	13.6^a^ (1.7)	14.6 (2.2)	*F*(2, 87) = 4.7	*p* = .01
Estimated IQ	106.1^a^ (14.9)	90.3^a,b^ (19.9)	105.0^b^ (14.5)	*F*(2, 87) = 7.7	*p* < .001
BPRS Total Score	28.4^a^ (4.2)	43.8^a,b^ (11.1)	32.7^b^ (7.7)	*F*(2, 87) = 29.6	*p* < .001
Positive Symptoms	5.1^a^ (0.4)	13.3^a,b^ (6.9)	5.7^b^ (1.7)	*F*(2, 87) = 41.1	*p* < .001
Negative Symptoms	3.1^a^ (0,3)	5.5^a,b^ (2.8)	3.4^b^ (1.0)	*F*(2, 87) = 17.7	*p* < .001
Disorganized Symptoms	5.2^a^ (1.4)	7.0^a^ (2.7)	6.2 (1.7)	*F*(2, 87) = 6.4	*p* = .003

Parentheses indicate standard deviations unless noted otherwise. *p*-values indicate differences in measures across diagnostic categories: schizophrenia probands (PSZ), controls (CTRL) and relatives of PSZ (REL). Paired superscripts indicate differences between groups for a given measure, *p* < .05. BPRS = Brief Psychiatric Rating Scale (24 item version).

### 2.2 Spatial working memory task, EEG acquisition and processing

**Fig. 1:**
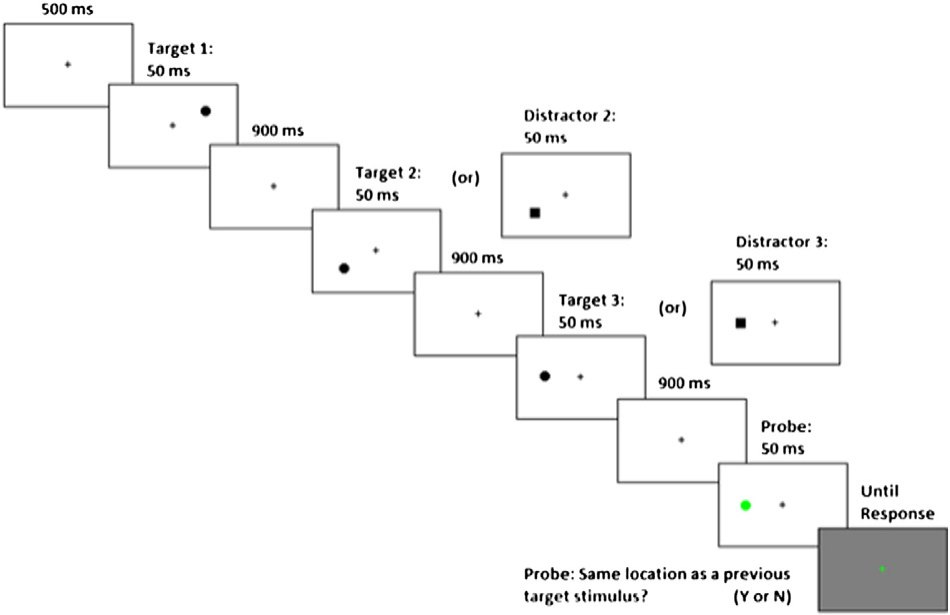
Participants were administered a spatial working memory task in which either two or three memory stimuli were sequentially presented on the screen. Each stimulus was presented at one of sixteen possible locations configured circularly around a central fixation. These stimuli were either “targets” (black circles) or “distractors” (black squares); a maximum of one distractor could appear per trial. After the memory stimuli were presented, a probe stimulus (a green circle) was subsequently presented, and participants were asked to indicate whether or not the probe stimulus appeared in the position of a previous *target* stimulus; if the probe appeared in the position of a previous distractor, the participants were instructed to respond “no.” The task included 2 two-stimulus blocks and 6 three-stimulus blocks of 36 trials each, presented in a pseudo-randomized order. Participants who performed at less than 60% accuracy on two-stimulus trials were excluded from behavioral and ERP analyses. Stimuli were presented in one of 16 locations on an invisible circle with a radius subtending a visual angle of 9.3 degrees. Stimuli subtended a visual angle of 1.6 degrees, and potential locations were separated by 22.5 degrees of arc around the circumference of the circle.

Participants were administered a spatial WM task derived from Park's (1997) delayed response task; see Fig. 1 for further description. EEG was recorded using a BioSemi Active-Two *AgCl* electrode system (BioSemi Inc., Amsterdam, The Netherlands). Recordings utilized a 128-channel, full scalp dense array sampled at 1024 Hz. Recordings were down-sampled offline to 512 Hz, high-pass filtered at 0.5 Hz, and transformed to a linked earlobe reference. Data were preprocessed using a custom independent component analysis (ICA) based method for ocular, muscular and cardiac artifact removal; see supplementary materials for details.

### 2.3 ERPs

To investigate whether memory stimuli were differentially processed during encoding, ERPs were computed for stimulus type (target vs. distractor). To examine neural processes associated with increasing WM load during encoding, we computed ERPs for order of stimulus presentation (first vs. second vs. third). Finally, to study neural processes associated with retrieval as modulated by encoding manipulations, we computed ERPs for probe location based on the type of encoding stimulus that appeared in the same location (“encoded type”: probe at previous target vs. previous distractor location vs. elsewhere), as well as probe location based on the sequential position of the encoding stimulus (“encoded order”: probe at first vs. second vs. third stimulus position vs. elsewhere). ERPs were time-locked to the relevant stimulus and epoched from −150 ms to 850 ms with stimulus onset designated as 0 ms; subject averages were low-pass filtered at 20 Hz for ERP component analysis.

ERP components of interest included the P1, N1, and a late positive potential (LPP) encompassing the P300 but extending as far as 850 ms after stimulus onset. P1 and N1 were measured by computing peak amplitudes between 100 and 175 ms and 125–225 ms respectively. The LPP was assessed by computing mean amplitudes within 50 ms time windows between 200 and 850 ms after stimulus onset. For each independent variable (stimulus type, order of stimulus presentation, probe location/stimulus type and probe location/presentation order), P1 and N1 were measured at electrode sites PO4 and PO8, and the LPP was measured at sites FC1, C2, and CP1. All reported findings are for these electrode sites because they contained the greatest component amplitudes.

### 2.4 Statistical analyses

To examine the effects of group status and task manipulations on participants' performance of the WM task, separate repeated measures ANOVAs were run for independent variables of number of trial stimuli (two vs. three), trial type (with vs. without distractor), probe location based on encoded stimulus type (probe at previous target vs. distractor location vs. elsewhere), and probe location based on presentation order of encoded stimulus (probe at first vs. second vs. third stimulus location vs. elsewhere); diagnostic group (CTRL, PSZ or REL) was included in each ANOVA as an additional factor.

ERP measures were analyzed using mixed model ANCOVAs. Separate ANCOVAs were run for independent variables of stimulus type, order of stimulus presentation, probe type, and probe order. Each model included as fixed factors the relevant independent variable, electrode site, diagnostic group (PSZ, REL or CTRL), and for the LPP, time window. Subject was included as a random factor; age and gender were included as covariates. Nearly all effects of electrode and time window were significant; those associated with group status and task manipulations are reported and with Greenhouse–Geisser (G-G) correction where appropriate. Post-hoc testing of significant ANCOVAs used Tukey's Honestly Significant Difference tests. In order to determine the relationship between ERP measures and behavioral performance, Pearson's correlations were computed for measures showing differences between CTRL and PSZ.

## 3 Results

### 3.1 Performance on the spatial working memory task

**Fig. 2:**
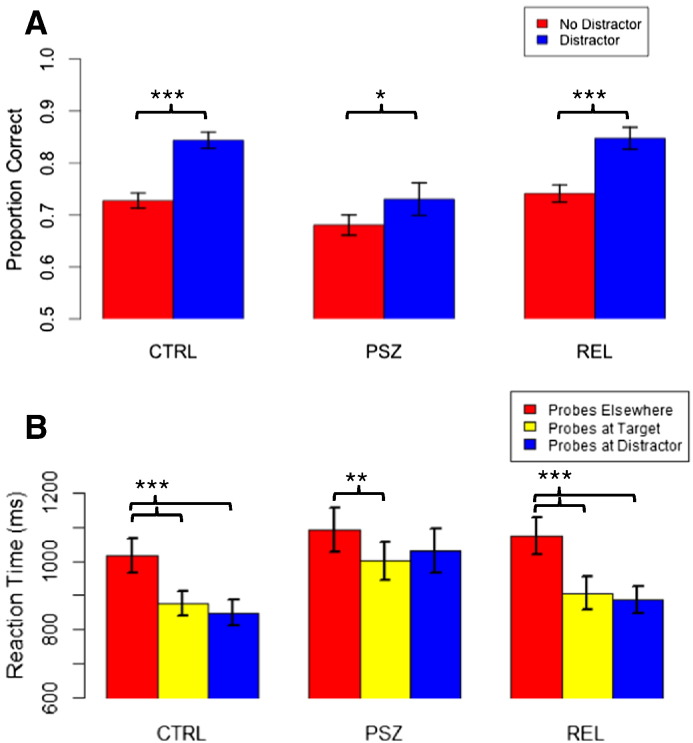
a) Proportion correct on No Distractor trials (red) versus Distractor trials (blue) for controls (CTRL), schizophrenia probands (PSZ), and relatives of PSZ (REL); CTRL and REL showed a markedly greater advantage in performance on Distractor trials than PSZ. b) Response reaction times to Probes at Previous Target positions, Previous Distractor positions, and Elsewhere for CTRL, PSZ and REL; CTRL and REL showed decreased reaction times to probes in Previous Distractor positions as well as Previous Target positions, whereas PSZ showed decreased reaction times only to probes in the position of a Previous Target stimulus. *p < .05, **p < .01, ***p < .001.

Behavioral results for the spatial WM task are presented in Table 2. ANOVAs revealed that groups differed in accuracy across task manipulations (number of stimuli: *F*(2,87) = 6.06, *p* = .003, *η*^*2*^_*p*_ = .12; trial type: *F*(2,87) = 7.17, *p* = .001, *b* = .14; probe location, encoded stimulus type: *F*(2,87) = 7.84, *p* = .001, *η*^*2*^_*p*_ = .15; probe location, presentation order of encoded stimulus: *F*(2,87) = 7.15, *p* = .001, *η*^*2*^_*p*_ = .14). In all cases, PSZ performed worse than CTRL (*p*s < .012, *d*s ≥ .71) and REL (*p*s < .005, *d*s ≥ .77); REL failed to differ from CTRL on behavioral indices. Effects of task manipulations on performance did not differ across groups, except for an observed interaction between group and trial type, *F*(2,87) = 3.44, *p* = .036, *η*^*2*^_*p*_ = .07. Here, PSZ benefitted markedly less from the presence of a distractor (*p =* .02, *d* = .40) than did CTRL and REL (*p*s < .001, *d*s ≥ 1.03; Fig. 2a). Examination of reaction times to probes revealed an interaction between group and probe location, *F*(3.56,149.70) = 2.51, *p* = .05, *η*^*2*^_*p*_ = .06. CTRL and REL were quicker to react to probes in previous distractor positions as well as previous targets (*p*s < .001, *d*s > .62), whereas PSZ showed faster reactions to probes in previous target positions (*p* = .005, *d* = .32) but only a trend for probes in previous distractor positions (*p* = .08, *d* = .20; Fig. 2b). Thus, CTRL and REL performed better than PSZ across all task conditions, and PSZ gained a lesser performance advantage on trials with distractors than did CTRL and REL.

**Table 2:** Proportion of trials correct on spatial working memory task.

	CTRL (*n* = 37)	PSZ (*n* = 23)	REL (*n* = 30)	*F*-value	*p*-value
Overall	.78^a^ (.08)	.71^a,b^ (.12)	.79^b^ (.09)	*F*(2, 87) = 7.1	*p* = .001
Two-Stimulus Trials	.81 (.09)	.75^b^ (.13)	.83^b^ (.10)	*F*(2, 87) = 3.8	*p* = .03
Three-Stimulus Trials	.78^a^ (.08)	.69^a,b^ (.12)	.78^b^ (.09)	*F*(2, 87) = 7.7	*p* < .001
No Distractor Trials	.73 (.09)	.68^b^ (.09)	.74^b^ (.09)	*F*(2, 87) = 3.2	*p* = .047
Distractor Trials	.84^a^ (.09)	.73^a,b^ (.15)	.85^b^ (.12)	*F*(2, 87) = 8.2	*p* < .001
Probe at Previous Target	.84 (.15)	.76^b^ (.17)	.86^b^ (.12)	*F*(2, 87) = 3.5	*p* = .03
Probe at Previous Distractor	.89^a^ (.10)	.72^a,b^ (.17)	.86^b^ (.18)	*F*(2, 87) = 9.6	*p* < .001
Probe Elsewhere	.64 (.17)	.62 (.15)	.65 (.16)	*F*(2, 87) = 0.3	*p* = .75
Probe at 1st Position	.78 (.20)	.72 (.20)	.82 (.09)	*F*(2, 87) = 2.3	*p* = .11
Probe at 2nd Position	.86^a^ (.12)	.74^a,b^ (.15)	.86^b^ (.14)	*F*(2, 87) = 5.8	*p* = .004
Probe at 3rd Position	.90^a^ (.08)	.75^a,b^ (.16)	.88^b^ (.13)	*F*(2, 87) = 10.4	*p* < .001

Parentheses indicate standard deviations unless noted otherwise. *p*-values indicate differences across diagnostic categories: schizophrenia probands (PSZ), controls (CTRL) and relatives of PSZ (REL). Proportions that share the same superscript for a given index (e.g., row) differ between groups, *p* < .05.

### 3.2 Neural responses during working memory encoding

#### 3.2.1 Target vs. distractor stimuli

ERPs were computed for target and distractor stimuli in an effort to investigate whether the relevance of stimuli affected neural responses. Early P1 responses over occipital brain regions showed a main effect of stimulus type (*F*(1,87) = 38.11, *p* < .001, *η*^*2*^_*p*_ = .30), demonstrating that target and distractor stimuli were differently encoded. Furthermore, LPP responses showed a significant interaction between stimulus type and time window, *F*(7.55,656.58) = 9.45, *p* < .001, *η*^*2*^_*p*_ = .10, suggesting temporally isolated discrimination between the stimulus types. P1 and LPP amplitudes were greater in response to distractors than targets (*p*s *< .*03, *d*s > .29). There was no main effect of group on encoding ERPs, and stimulus type failed to interact with group for the ERP amplitudes. Thus, P1 and LPP responses suggest successful discrimination of target versus distractor stimuli across all diagnostic groupings.

#### 3.2.2 Stimulus order (i.e., working memory load)

ERPs to stimuli were averaged based on their order of presentation (first, second, or third) to investigate neural responses related to increasing WM load at encoding. Early N1 responses over occipital regions showed effects of stimulus order (*F*(1.55,135.04) = 12.61, *p* < .001, *η*^*2*^_*p*_ = .13), where responses to both second (*p* < .001) and third (*p* < .001) stimuli were significantly greater (more negative) than those to first stimuli; similar patterns were observed across diagnostic groups. The LPP over central midline sites showed a significant interaction between presentation order and time window, *F*(12.38,1077.42) = 10.58, *p* < .001, *η*^*2*^_*p*_ = .11. Examination of the group distributions of mean amplitude across time window suggested differential patterns of responses across groups (Fig. S2). Because PSZ showed notably deviant waveforms from CTRL, within group ANCOVAs of LPP responses were conducted and confirmed that presentation order by time window interactions were observed for CTRL, *F*(8.36,300.82) = 5.67, *p* < .001, *η*^*2*^_*p*_ = .14, and REL, *F*(7.96,230.74) = 5.89, *p* < .001, *η*^*2*^_*p*_ = .17, but not PSZ, *F*(8.07,177.52) = 1.59, *p* = .13, *η*^*2*^_*p*_ = .07. Thus, LPP responses indexed WM load in CTRL and REL, but not in PSZ; early N1 responses, however, mirrored increases in WM load for all participants, regardless of diagnostic status.

### 3.3 Neural responses during retrieval from working memory

#### 3.3.1 Stimulus type (previous target versus previous distractor)

**Fig. 3:**
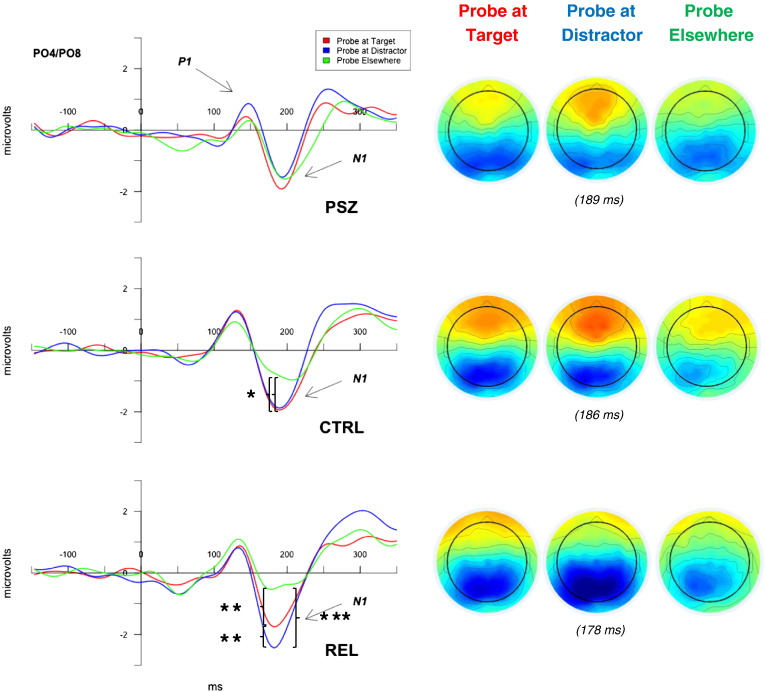
*Target* vs. *Distractor Effects on Retrieval N1*. N1 ERPs (left panel) and scalp topographies (right panel) in response to probes at previous target locations (red), previous distractor locations (blue), elsewhere (green). Waveforms are averaged across electrodes PO4 and PO8. For schizophrenia probands (PSZ), N1 responses to probes failed to differentiate whether the location was associated with a target, distractor, or neither at encoding, while N1 responses at retrieval for controls (CTRL) and relatives (REL) groups did differentiate the encoding status of the spatial location. REL alone showed larger N1 amplitudes to distractors compared to targets. *p < .05, **p < .01, ***p < .001.

In order to investigate neural responses to probes at previously encoded locations, ERPs to probe stimuli were averaged based on whether they appeared in the position of a previous target, distractor, or elsewhere (“encoded type”). Early N1 responses over occipital brain regions showed an interaction between diagnosis and encoded type, *F*(4174) = 8.17, *p* < .001, *η*^*2*^_*p*_ = .16 (Fig. 3). While a main effect of encoded type was observed for CTRL, *F*(2,72) = 4.09, *p* = .02, *η*^*2*^_*p*_ = .10, and REL, *F*(2,58) = 24.56, *p* < .001, *η*^*2*^_*p*_ = .46, PSZ showed no such effect, *F*(2,44) = 0.68, *p* = .51, *η*^*2*^_*p*_ = .03. CTRL's N1 responses to probes at previous targets (*p =* .04, *d* = .26) as well as to probes at previous distractors (*p* = .04, *d* = .25) were larger than those to probes elsewhere; in REL, N1 responses to probes at distractors were greater than probes at targets (*p* = .003, *d* = .43), which in turn were greater than probes elsewhere (*p* = .002, *d* = .40). Thus, early N1 responses to probe stimuli for PSZ fail to be affected by prior encoding information, while such responses in REL and CTRL do differentiate previously encoded spatial locations.

**Fig. 4:**
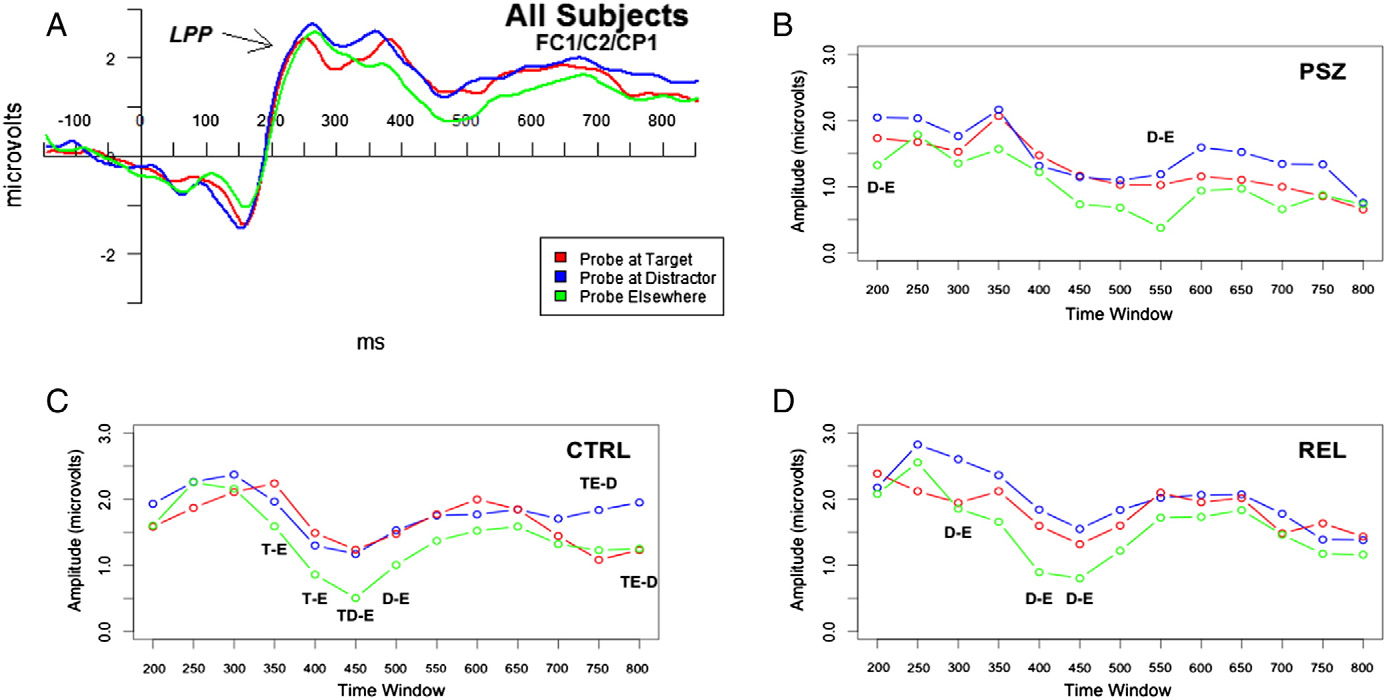
LPP responses to probes at previous target location (red), probes at previous distractor location (blue), and probes elsewhere (green). Waveforms are derived from averages across electrodes FC1, C2 and CP1 for a) all subjects, b) people with schizophrenia (PSZ), c) controls (CTRL), and d) relatives of PSZ (REL). The x-axis represents the beginning of each 50 ms time window. Significant differences (*p* < .05) in time window mean amplitudes between probes at previous target locations (T), previous distractor locations (D), and elsewhere (E) are indicated with a dash between labels for differing conditions (e.g., T − E = difference between target and elsewhere). PSZ fail to show differences between conditions in the 350 ms to 550 ms time window, in contrast to CTRL and REL.

Late central midline LPP responses showed an interaction between encoded type and time window, *F*(12.58, 1094.03) = 2.31, *p* = .006, *η*^*2*^_*p*_ = .03; Fig. 4a). Examination of the waveforms suggested possible differences in late responses across diagnostic groups (Fig. 4b–d); as such, within-group analyses were performed due to what appeared to be deviance in the PSZ and REL waveforms as compared to that of CTRL. Within-group ANCOVAs confirmed that significant encoded type-by-time-window interactions were observed for CTRL, *F*(9.85,354.53) = 2.77, *p* = .003, *η*^*2*^_*p*_ = .07, but neither REL, *F*(8.33,241.48) = 1.39, *p* = .20, *η*^*2*^_*p*_ = .05, nor PSZ, *F*(7.37,162.05) = 0.75, *p* = .64, *η*^*2*^_*p*_ = .03. Fig. 4 illustrates that RELs' and PSZs' late responses to probe stimuli failed to differentiate between the previously encoded locations as compared to CTRL. Interestingly, LPP amplitudes to probes elsewhere were predictive of overall behavioral performance for PSZ alone between 400 ms and 450 ms post-probe (PSZ: *r* = −.43, *p* = .04; CTRL: *r* = .06, *p* = .74; REL: *r* = −.04, *p* = .83) as well as between 450 ms and 500 ms post-probe (PSZ: *r* = −.45, *p* = .03; CTRL: *r* = .01, *p* = .98; REL: *r* = −.04, *p* = .83). Thus, for PSZ, greater LPP responses to probes in task-irrelevant locations, perhaps reflecting errant recognition, predicted poorer performance in the 400 ms to 500 ms post-probe time window.

#### 3.3.2 Encoded stimulus order (first vs. second vs. third stimulus)

**Fig. 5:**
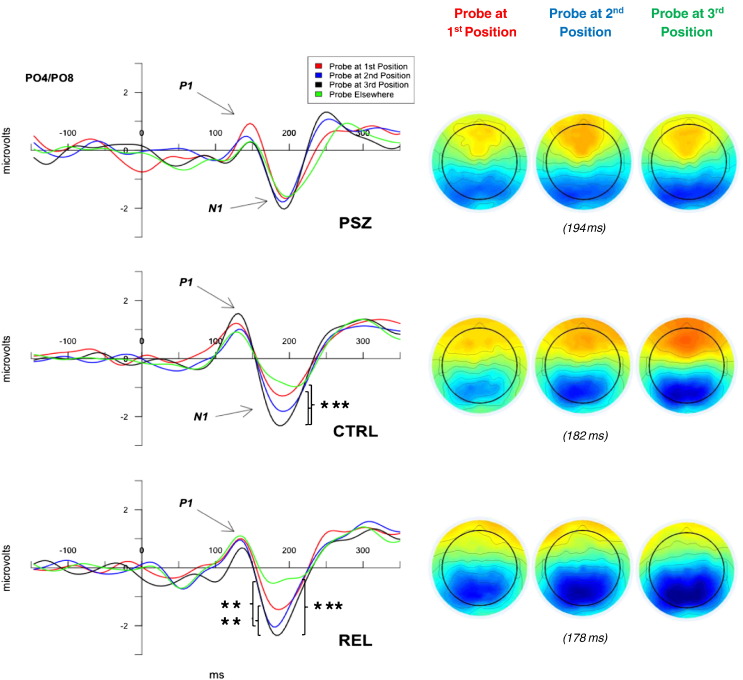
*Stimulus Order Effects on Retrieval N1*. N1 ERPs (left panel) and scalp topographies (right panel) to probes at previous 1st stimulus location (red), 2nd stimulus location (blue), 3rd stimulus location, (black), and elsewhere (green). Waveforms are derived from averages across electrodes PO4 and PO8 for people with schizophrenia (PSZ), controls (CTRL), and relatives of PSZ (REL). N1 responses differentiated probes presented at 3rd stimulus locations from probes at 1st stimulus locations and probes elsewhere for CTRL and REL, but not for PSZ. *p < .05, **p < .01, ***p < .001.

In order to investigate neural responses to probe positions relative to the order of stimulus presentation, ERPs to probe stimuli were averaged based on whether they appeared in the position of a first, second, third stimulus, or elsewhere (“encoded order”). Early N1 responses recorded over occipital regions revealed a diagnosis-by-encoded order interaction, *F*(5.03,218.92) = 2.47, *p* = .03, *η*^*2*^_*p*_ = .05. Post-hoc testing revealed that CTRL and REL showed greater N1 responses to probes in third stimulus positions than those in first positions and elsewhere (*p*s < .009, *d*s > .45), whereas PSZ demonstrated no effect of probe order (Fig. 5). Thus, CTRL and REL showed increasing N1 amplitudes to probes based on their position relative to the presentation order of the encoding stimuli, whereas PSZ failed to show such an effect. In addition, N1 responses to probes in the position of second stimuli predicted behavioral performance on trials featuring distractors in REL (*r* = −.43, *p* = .02) but neither CTRL (*r* = −.15, *p* = .39) nor PSZ (*r* = .02, *p* = .95). Thus, more robust N1 responses to probes in REL reflected more effective differentiation of previously encoded relevant and irrelevant locations.

## 4 Discussion

We administered a delayed response spatial WM task to PSZ, their first-degree biological relatives, and healthy controls during which we recorded EEG. We analyzed behavioral data and event-related neurophysiological responses in order to clarify the nature of spatial WM deficits in PSZ and their biological relatives (see Table 3 for a summary of findings). PSZ exhibited WM performance deficits and smaller neural responses as compared to controls. In particular, early posterior responses (N1) in PSZ recorded during retrieval failed to differentiate previously encoded locations while such responses in CTRL and REL varied depending on encoding status of the location.

**Table 3:** Summary of electrophysiological ANCOVA findings.

Component Amplitude	Differences in Stimulus Type or Order Condition	Group × Stimulus Condition interaction effect	Time window × Stimulus Condition interaction effect
**Encoding: Stimulus type and order of stimulus**			
P1	Distractor > Target	n.s.	
N1	Third, Second > First	n.s.	
LPP	Distractor > Target	n.s.	Significant: - Distractors > Targets for 200–250 ms, 300-600 ms - Targets > Distractors for 800–850 ms
LPP	n.s. (First, Second, Third)	n.s.^a^	Significant: - Second and Third stimuli > First for 200–450 ms - Third stimulus < First and Second after 700 ms

**Retrieval: Probe location based on encoded stimulus type and encoded stimulus order**			
N1	Target, Distractor > Elsewhere	Significant: - CTRL: Target, Distractor > Elsewhere - PSZ: No differences across locations - REL: Distractor > Target > Elsewhere	
N1	Third > Second, First	Significant: - CTRL: Third > First - PSZ: No difference across order - REL: Third > First	
LPP	Target, Distractor > Elsewhere	n.s.^b^	Significant: - Distractors > Targets for 250–350 ms - Distractors > Elsewhere for 300-650 ms, 700–850 ms - Targets > Elsewhere for 350–600 ms - Elsewhere > Targets for 250–300 ms

Indicated effects are significant at *α* = .05. All main effects of group were non-significant. Superscripts indicate analyses where within-group ANCOVAs were performed on the basis of observable differences between group waveforms despite a lack of a significant group by manipulation interaction. For retrieval results, “Target” refers to probes in the position of an encoding target stimulus, “First” to probes in the position of the first encoding stimulus, “Elsewhere” to probes that did not appear in the position of an encoding stimulus, etc.

a The stimulus order × time window interaction was significant for CTRL, REL.

b The encoded stimulus order × time window interaction was significant for CTRL alone.

### 4.1 Working memory performance & distraction in schizophrenia

PSZ performed more poorly on the WM task than CTRL and REL across all task conditions. Such performance deficits are consistent with the widespread WM deficits typically observed in PSZ (Forbes et al., 2009, Lee and Park, 2005). Notably, all participant groups showed higher accuracy rates on trials that featured a distractor versus those that did not. Together with the observed reduction in reaction times to probes in the position of previous distractors, these findings suggest that participants benefitted from successfully encoding distractor stimuli. However, the interaction effects between group and trial type as well as group and reaction time indicate that PSZ were less able to use distractors to their advantage as compared to CTRL and REL. Hence, though PSZ are capable of successfully differentiating distracting stimuli when they are not overly salient (Erickson et al., 2015, Erickson et al., 2014, Gold et al., 2006, Gray et al., 2014, Hahn et al., 2010), they do so less efficiently than healthy controls; such a result is consistent with fMRI studies demonstrating inefficiency of cognitive processing in PSZ (Cairo et al., 2006, Schlӧsser et al., 2008). Though PSZ gained some advantage from encoding distractors, their relative improvement was not as great as that observed in REL and CTRL.

### 4.2 Neural responses during working memory

#### 4.2.1 Abnormal early posterior brain responses at retrieval in schizophrenia reflect a failure to utilize encoding information

During retrieval, CTRL and REL demonstrated enhanced N1 responses to probe stimuli in the positions of previous stimuli, while PSZ showed no such response. N1 has been well established as an index of orientation to task-relevant locations (Luck et al., 1990); Dias et al. (2011) have reported similar modulations in PSZ and controls of N1 amplitude to probes based upon the prior cue. In the present study we found a failure in PSZ to modulate N1 responses to probes based on their spatial relation to encoding stimuli. It is possible that abnormalities observed during retrieval reflect failures to successfully encode stimuli in the first place; however, neural responses (namely, the P1 and P300) suggest some successful neural discrimination between differing stimuli during encoding. These findings are consistent with PSZs' behavioral performance and previous findings of preserved function of the parvocellular visual pathway in PSZ (e.g., Dias et al., 2011). Neural abnormalities at retrieval in PSZ may likewise reflect a failure to maintain encoded information in a manner that would affect early processing of stimuli at retrieval, consistent with previously reported maintenance deficits in PSZ (Fleming et al., 1997, Haenschel et al., 2007, Lee and Park, 2005). Furthermore, REL showed augmented N1 responses to probes at distractor locations, suggesting a potential sensitivity of biological relatives to distractor stimuli. Similarly, N1 responses to probes showed greater modulation by the presentation order of the stimuli in REL than CTRL, suggesting that the “traces” of these relevant positions are more clearly delineated. However, REL show LPP responses similar to PSZ for probe stimuli during retrieval. Functional MRI studies have shown similar activation deficits in PSZ and REL; however, REL also demonstrate increased activations as compared to CTRL in certain brain regions that have been posited to represent compensatory responses (reviewed in Zhang et al., 2016). The presently reported abnormalities in the modulation of early visual processes indexed by the N1 in REL for WM retrieval may similarly represent a compensatory neural mechanism that facilitates intact performance by biological relatives of PSZ on this spatial WM task featuring simple stimuli.

#### 4.2.2 Predicting behavioral performance with ERP measures

Neural responses to probe stimuli proved most predictive of behavioral performance for PSZ and REL, but were not predictive for CTRL. PSZ performance related to LPP responses to probes that appeared in a location not previously occupied by an encoding stimulus, suggesting that PSZ who showed the strongest neural recognition of probes in a novel location exhibited errant probe location recognition. REL performance was predicted by N1 responses to probes in the second stimulus position. These associations were not significant for CTRL, suggesting that retrieval processes are particularly important to efficient WM ability in PSZ and REL, and that neural function during spatial WM retrieval may be the critical determinant of WM dysfunction in the disorder.

In conclusion, the present study demonstrated neural deficits in PSZ during both encoding and retrieval. REL showed encoding responses comparable to CTRL, in addition to preserved and even heightened modulations of early responses to probes during retrieval despite late responses similar to PSZ. This notable difference between PSZ and REL during retrieval may suggest a compensatory mechanism for REL allowing them to perform at the level of CTRL. Consistent with this notion, both PSZ's and REL's task performance was predicted by neural responses during retrieval, emphasizing the importance of retrieval processes to WM function in PSZ and their unaffected relatives.

## Role of Funding Source

The funders had no role in the study design, data collection and analysis, decision to publish, or preparation of the manuscript.

## Contributors

PAL programmed the behavioral task, collected much of the data, designed and performed data analyses and wrote the submitted manuscript. SSK wrote the base scripts used in processing the data and from which the analysis scripts were derived, reviewed the manuscript, and offered commentary. SRS designed the behavioral task and experiment, secured funding for the project, oversaw the recruitment and clinical assessment of participants, advised the data analyses and writing of the manuscript, and carried out editing of the submission.

## Conflict of Interest

The authors have no conflict of interest.
