# Supplementary material for: Impaired retrieval processes evident during visual working memory in schizophrenia
Source: Schizophr Res Cogn. 2016 Aug 11;5:47–55. doi: 10.1016/j.scog.2016.07.002 (PMC5514301; doi:10.1016/j.scog.2016.07.002)
Supplement: Supplementary materials [file mmc1.docx]

**Supplemental Methods**

**Participants**

**Clinical measures.** Enrolled participants underwent clinical assessments that included the Structured Interview for DSM-IV-TR, Patient Edition (SCID; First, Spitzer, Gibbon, & Williams, 2002), the Scale for the Assessment of Positive Symptoms and Scale for the Assessment of Negative Symptoms (SAPS and SANS; Andreasen, 1984a, 1984b), and the Brief Psychotic Rating Scale (BPRS; Overall & Gorham, 1962) with all measures being administered by a trained and supervised B.A. or M.A. level research assistant or M.A. or Ph.D. level clinicians. All participants were subjected to a consensus diagnosis process, in which two or more Ph.D. clinicians or advanced doctoral students reviewed each participant’s interview data and other available study materials to form a jointly agreed upon diagnosis.

**Inclusion and exclusion criteria.** To be included as a PSZ, participants were required to have a diagnosis of schizophrenia or schizoaffective disorder, depressed type as determined by SCID diagnosis confirmed by consensus review. Exclusion criteria for PSZ included being over 60 years old, lacking first degree biological relatives, being an adoptee, IQ < 70, substance dependence in the past 6 months, substance abuse in the two weeks prior to study participation, or more than three substantial uses of inhalants. PSZ were further excluded for a past skull fracture or loss of consciousness of 30 minutes or more, past electroconvulsive therapy, a current or past diagnosis of epilepsy, a history of multiple seizures in adulthood, a documented seizure in the 6 months prior to participation, a history of strokes, other neurological conditions, possessing non-removable hearing aids, legal blindness, having an uncorrected lazy eye, compromised visual acuity unable to be corrected to normal, other visual conditions that compromise vision, and other general medical conditions that made participation impossible and/or substantially affected brain functioning. CTRL had all the same exclusion criteria as PSZ, except that CTRL could participate without first-degree relatives; in addition, a past or current diagnosis of schizophrenia, schizoaffective disorder, any other psychotic disorder, bipolar affective disorder (I or II), learning disability, a past or current depressive episode, and/or a family history of psychotic symptoms or bipolar disorder precluded participation as a CTRL. The only exclusion criteria for participation as a REL were visual conditions / acuity that could not be corrected to normal and/or general medical conditions that prevented participation.
**EEG Recording and Preprocessing**
 Data were preprocessed using a custom independent component analysis (ICA) based method for ocular, muscular and cardiac artifact removal; see supplementary materials for details.. Data were visually inspected and bad electrodes and time segments judged to contain significant signal noise other than brain activity were excluded. Continuous recordings were epoched into 1000 ms stimulus-locked epochs. The epoched data were transformed into the frequency domain and plotted highlighting low-frequency and high-frequency signal in sequence, with extreme epochs being removed based on visual inspection. Data were retransformed into the time domain, and final ICs were identified as either primarily brain or artifact based on visual inspection; the denoised signal was reconstituted using those ICs dominated by brain signal. Participants for whom greater than 35% of their EEG data were contaminated by artifact were excluded from behavioral and ERP analyses.
**ERP Components** ERP components of interest included the P1, N1, and a late positive potential (LPP) encompassing the P300 but extending as far as 850 ms after stimulus onset. The P1, which is most commonly analyzed in the context of amplitude modulation dependent on the focus of spatial attention (Luck et al., 1990; Luck et al., 1994), also shows amplitude modulation indexing the suppression of task-irrelevant information, with greater P1 amplitudes being elicited by task-relevant stimuli (Finnigan et al., 2011; Luck & Hillyard, 1995). The N1, like the P1, reflects “gain control” of sensory processing, with greater amplitudes observed at attended versus unattended locations. The N1 has further demonstrated increased amplitudes with the orientation of attention to task-relevant locations (Luck et al., 1990), and has also been shown to index processes of discrimination, with larger responses being observed as discrimination demands increase (Vogel & Luck, 2000). The P300 is a complex component shown to be modulated by many different task manipulations. It is most often connected with probabilistic evaluations in the form of increased amplitude associated with novelty processing (the traditional P300 and P3a) or the occurrence of expected but infrequent events (the P3b) (Polich, 2012).
**Statistical Analyses**
 To understand the effect of the relative spatial distribution of stimuli on behavioral performance, a mixed effects logistic regression model was run with total distance between stimuli divided by the number of stimuli per trial (continuous) entered as a predictor of trial performance; diagnostic group (PSZ, REL or CTRL) was also included as a moderator variable to examine whether the effects of these manipulations differed across groups. Significance of predictor coefficients was assessed using Wald *Z*-tests.

**Supplemental Results**

**Performance on the Spatial Working Memory Task**
 **Effects of task manipulations on performance.** A main effect of number of stimuli per trial was observed, *F*(1, 87) = 42.77, *p* < .001, *η^2^_p_*=.33, wherein participants performed better on two-stimulus trials than three-stimulus trials (*p* < .001, *d* = .41). We likewise observed a main effect of trial type, *F*(1, 87) = 84.96, *p* < .001, *η^2^_p_*=.49, where better performance was observed on distractor trials than no-distractor trials, *p* < .001, *d* = .87. Main effects of probe location, encoded stimulus type, *F*(1.60, 138.78) = 51.04, *p* < .001, *η^2^_p_*=.37, and probe location, presentation order of encoded stimulus, *F*(1.39, 121.10) = 20.04, *p* < .001, *η^2^_p_*=.19, were also observed; here, participants performed significantly better on trials with probes at a previous stimulus location than a probe elsewhere (*p*s < .001, *d*s > 1.19), and performed better on trials where probes appeared in the position of later stimuli as compared to earlier stimuli (*p*s < .001, *d*s > .34).
 Examination of the proportions of correct trials in Table 2 reveals PSZ’s notable failure to improve performance from trials where the probe appeared in the position of the first stimulus to trials with the probe in the third stimulus position as compared to REL and CTRL. Thus, separate repeated measure ANOVAs were conducted within each diagnostic category, confirming significant main effects of probe order in CTRL, *F*(1.19, 43.01) = 12.61, *p* < .001, *η^2^_p_*=.26, and REL, *F*(1.48, 42.85) = 8.27, *p* = .002, *η^2^_p_*=.22, but not PSZ, *F*(1.49, 32.80) = 1.44, *p* = .25, *η^2^_p_*=.06. In both CTRL and REL, performance on trials where probes appeared in the position of the first stimulus was worse than on trials with probes in the second stimulus position (*p*s < .04, *d*s > .31) as well as trials with probes in the third stimulus position (*p*s < .001, *d*s > .49). Thus, CTRL and REL showed significantly better performance for probes in the positions of more recent stimuli, whereas PSZ did not.
 In order to examine the effect of spatial spread of visual stimuli encoded into working memory we examined distances between stimuli, a continuous variable, and its interaction with group status on behavioral performance. A mixed effects logistic regression analysis was conducted with spatial spread, diagnostic status and their interaction entered as predictors for performance accuracy. The analysis revealed a main effect of spatial spread (*β* = -.20, *SE* = .07, *p* = .005), such that greater overall distance between stimuli predicted poorer performance accuracy; no interaction between diagnostic status and spatial spread was observed. Thus, a wider spatial distribution of stimuli made trials more difficult. There was also a diagnostic group main effect where being a PSZ predicted a decrease in accuracy (*β_difference_* = -.42, *SE* = .14, *p* = .003) as compared to CTRL. **Neural Responses During Working Memory Encoding** **Target and distractor stimuli.** ERPs were computed for target and distractor stimuli in an effort to investigate whether the relevance of stimuli affected neural responses. ERP amplitude measures were subjected to mixed model ANCOVAs with fixed factors of stimulus type, electrode site and diagnostic group. Early neural responses over occipital brain regions showed main effects of stimulus type (P1: *F*(1, 87) = 38.11, *p* < .001, *η^2^_p_*=.30) as did the late neural response over central midline brain regions (LPP: *F*(1, 87) = 19.11, *p* < .001, *η^2^_p_*=.18) demonstrating that target and distractor stimuli were differently encoded. Furthermore, LPP responses showed a significant interaction between stimulus type and time window, *F*(7.62, 662.73) = 8.77, *p* < .001, *η^2^_p_*=.09, suggesting differences in LPP responses to the two stimulus types were confined to certain time windows. P1 and LPP amplitudes were greater in response to distractors than targets (*p < .*001, *d*s > .29). There was no main effect of group on encoding ERPs, and stimulus type failed to interact with group for the ERP amplitudes. However, inspection of waveforms (Figure S1) suggested a marked separation between LPP responses to targets versus distractors in REL alone beginning at 400 ms post-stimulus. Within-group ANCOVAs of LPP amplitude did indeed reveal a main effect of stimulus type reflective of larger LPP responses to distractor stimuli in REL, *F*(1, 29) = 19.78, *p* < .001, *η^2^_p_*=.41, but not in CTRL*, F*(1, 36) = 2,67, *p* = .11, *η^2^_p_*=.07, nor PSZ, *F*(1, 22) = 2.21, *p* = .15, *η^2^_p_*=.09. However, all groups showed stimulus type by time window interactions, suggesting some differentiation between stimulus types regardless of diagnostic status. Thus, P1 and LPP responses suggest successful discrimination of target versus distractor stimuli across all diagnostic groupings, though the LPP suggests this may be stronger in REL.
 **Stimulus order (i.e., working memory load).** Within group ANCOVAs of P1 responses confirmed that a main effect of stimulus order was observed in PSZ, *F*(2, 44) = 3.64, *p* = .03, *η^2^_p_*=.14, but not in CTRL, *F*(2, 72) = 0.67, *p* = .52, *η^2^_p_*=.02, nor REL, *F*(2, 58) = 0.10, *p* = .90, *η^2^_p_*<.01. In PSZ, P1 amplitudes to first stimuli were greater than those to second stimuli (*p* = .03, *d* = .54), whereas responses to third stimuli fell in between; as such, the effect of stimulus order on the P1 in PSZ may reflect a sensitivity to initial stimuli rather than an indexing of WM load.
**Neural Responses During Retrieval from Working Memory**

**Encoded stimulus order (first vs. second vs. third stimulus).** Late responses over central midline sites showed an effect of probe order (LPP: *F*(2.72, 236.53) = 6.42, *p* < .001, *η^2^_p_* =.07); LPP responses to probes elsewhere were less than those to probes in second stimulus positions (*p* < .001, *d* = .24) as well as those to probes in third stimulus positions (*p* = .003, *d* = .21). In addition, a probe order-by-time window interaction was observed, *F*(17.60, 1531.46) = 1.89, *p* = .01, *η^2^_p_*=.02. Thus, late responses at retrieval differentiate the order of stimuli presented at encoding. The order effects on the LPP at retrieval did not differ across diagnostic groups (Figure S3).

**A priori diagnostic group comparisons.** Although diagnostic group interacted with task conditions to yield abnormal neural functions during working memory in PSZ, there were no main effects of diagnosis observed in any of our ANCOVA analyses. We had hypothesized neural abnormalities in PSZ and REL as compared to controls and thus performed *a priori* Tukey comparisons for amplitude measures for each component / task manipulation pairing. None of these comparisons showed differences between the diagnostic groups (all *p*s > .05).

**Supplemental Discussion**

**Neural Responses during Working Memory** **Abnormal early posterior brain responses at retrieval in schizophrenia reflect a failure to utilize encoding information.** The early posterior P1 as well as N1 components have been shown to be sensitive to a number of manipulations in tasks probing visual WM, including frequency / expectation of stimuli, with greater amplitudes being observed for infrequent and thus unexpected stimuli (Dias et al., 2011), as well as for task relevant versus task irrelevant stimuli (Rutman, Clapp, Chadick & Gazzaley, 2010). We found that P1 responses successfully discriminated distractor stimuli from target stimuli during encoding for all participants, and N1 responses indexed increasing WM load across diagnostic groups. Thus, early attentional and discriminative processes during encoding of simple stimuli into visual spatial memory as indexed by the P1 and N1 are apparently undisturbed in PSZ and REL.
 **Increases in the number of items in working memory are reflected in increases in late positive brain responses.** Increasing WM load during encoding resulted in increasing LPP amplitudes in the P300 time window for controls and REL, but not PSZ. While Haenschel and colleagues (2007) have reported increases in P1 and P300 (P3a) amplitudes with increasing WM load during encoding for healthy controls, more frequently reported is a decrease in P300 amplitude as WM load increases (Polich, 2012). However, the vast majority of these evaluations examine varying WM load across trials, whereas our paradigm investigates the sequential increase of load within a trial, for which increasing P300 amplitude appears to reflect increasing load. In regards to this sequential increase in load, our task is similar to the standard digit span task, on which people with schizophrenia have been found to show performance deficits (Conklin et al., 2000). Furthermore, P300 amplitudes have been shown to increase with increasing WM load at lower digit loads (e.g., from two to four digits) in healthy individuals (Lefebvre, Marchand, Eskes & Connolly, 2005), consistent with the increases in P300 amplitude with increasing load we observed in our task. The lack of significant increases in P300 amplitude to successive trial stimuli for PSZ suggests difficulties in encoding these stimuli into WM as compared to CTRL, while stimulus encoding in REL is preserved.

**References for Supplemental Materials**

Andreasen, N. C. (1984a). *Scale for the assessment of negative symptoms (SANS).* Iowa City: University of Iowa.

--- (1984b). *Scale for the assessment of positive symptoms (SAPS).* Iowa City: University of Iowa.

Finnigan, S., O’Connell, R. G., Cummins, T. D. R., Broughton, M., & Robertson, I. H. (2011). ERP measures indicate both attention and working memory encoding decrements in aging. *Psychophysiology, 48*, 601-611.

First, M. B., Spitzer, R. L., Gibbon, M., & Williams, J. B. W. (2002). *Structured clinical interview for DSM-IV axis I disorders, research version, patient edition.* New York: Biometrics Research, New York State Psychiatric Institute.

Lefebvre, C. D., Marchand, Y., Eskes, G. A., & Connolly, J. F. (2005). Assessment of working memory abilities using an event-related brain potential (ERP)-compatible digit span backward task. *Clinical Neurophysiology, 116*, 1665-1680.

Luck, S. J., Heinze, H. J., Mangun, G. R., & Hillyard, S. A. (1990). Visual event-related potentials index focused attention within bilateral stimulus arrays: II. Functional dissociation of P1 and N1 components. *Electroencephalography & Clinical Neurophysiology*, *75*(6), 528-542.

Luck, S. J., & Hillyard, S. A. (1995). The role of attention in feature detection and conjunction discrimination: an electrophysiological analysis. *International Journal of Neuroscience, 80*, 281-297.

Luck, S. J., Hillyard, S. A., Mouloua, M., Woldorff, M. G., Clark, V. P., & Hawkins, H. L. (1994). Effects of spatial cuing on luminance detectability: psychophysical and electrophysiological evidence for early selection. *Journal of Experimental Psychology, 20*(4), 887-904.

Overall, J. E., & Gorham, D. R. (1962). The brief psychiatric rating scale. *Psychological Reports*, *10*, 799-812.

Polich, J. (2012). Neuropsychology of P300. In S. J. Luck & E. S. Kappenman (Eds.), *The* *oxford handbook of event-related potential components* (159-188). New York: Oxford University Press.

Rutman, A. M., Clapp, W. C., Chadick, J. Z., & Gazzaley, A. (2010). Early top-down control of visual processing predicts working memory performance. *Journal of Cognitive Neuroscience, 22,* 1224-1234.

Vogel, E. K., & Luck, S. J. (2000). The visual N1 component as an index of a discrimination process. *Psychophysiology, 37,* 190-203.


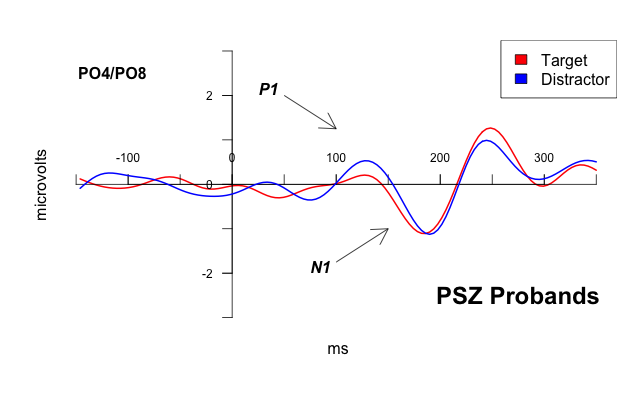

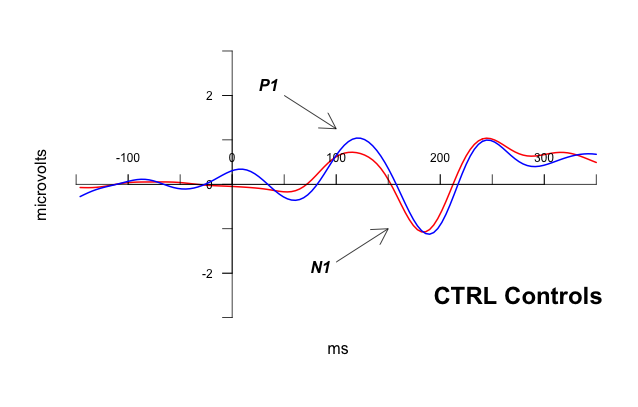

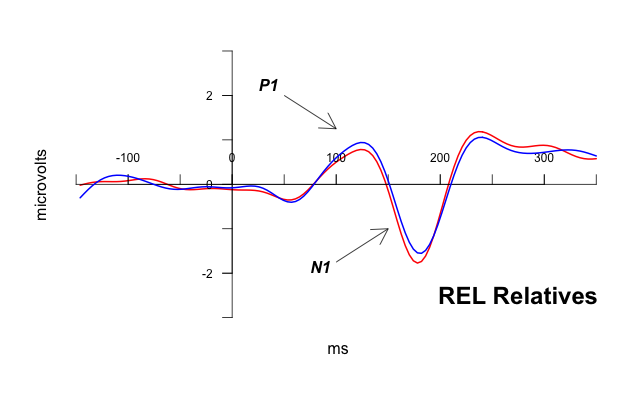


*Figure S1. Stimulus Type Effects on Encoding ERP Components*. P1 & N1 ERPs (left panel) and LPP ERPs (right panel) to target stimuli (red), and distractor stimuli (blue) for schizophrenia probands (PSZ), controls (CTRL), and relatives of PSZ (REL). P1 & N1 waveforms are derived from averages across electrodes PO4 and PO8; LPP waveforms are averages across electrodes FC1, C2, CP1. P1 responses were larger to distractor stimuli than target stimuli across all diagnostic groupings. LPP responses showed a main effect of stimulus type for REL alone, but all groups showed significant interactions between stimulus type and time window. **p* < .05, ***p < .01, ****p*<.001.*

**­*****

******


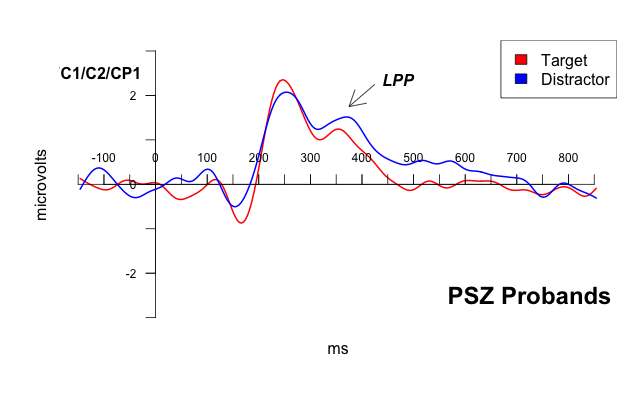

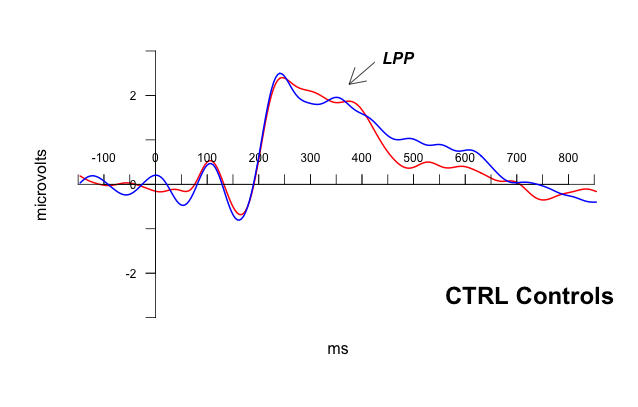

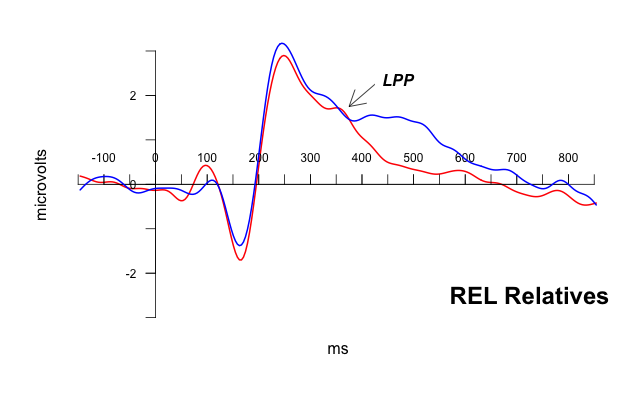


******

**­*****

**PSZ**

**PSZ**

**CTRL**

**CTRL**

**REL**

**REL**


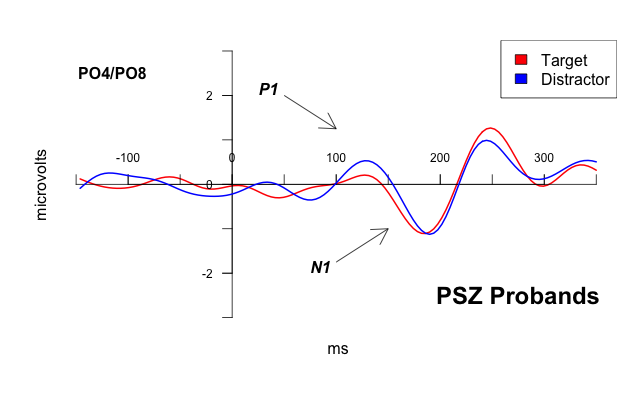

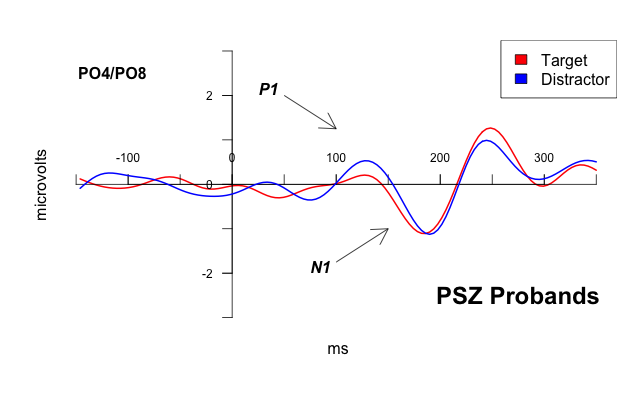


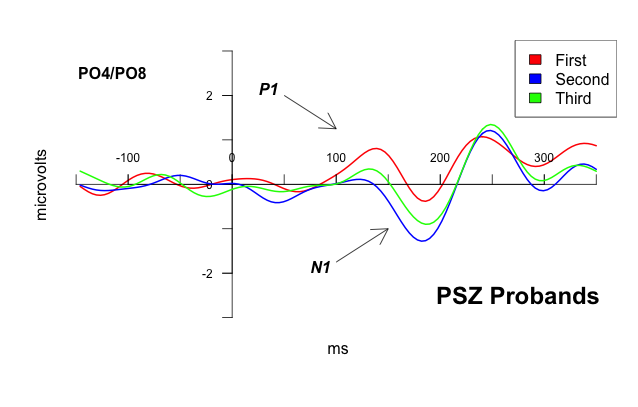

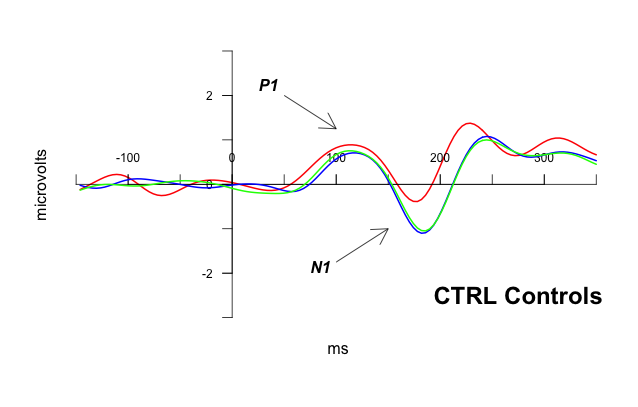

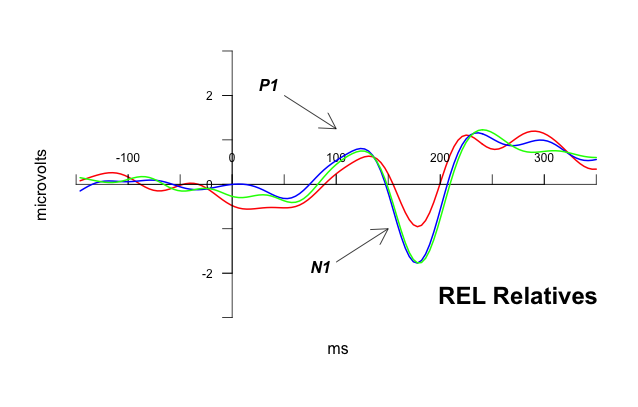

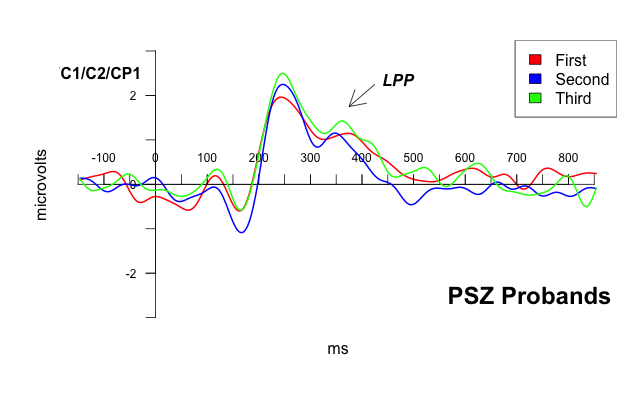

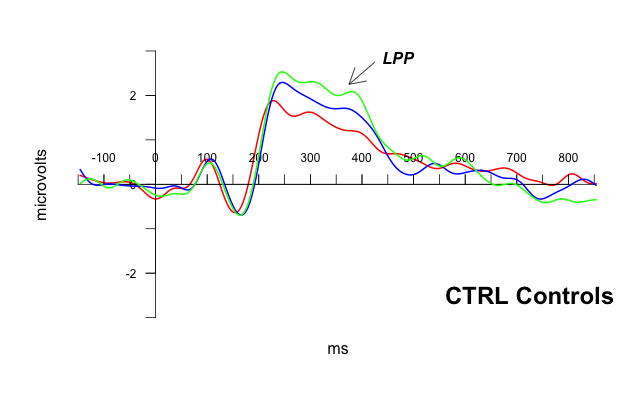


*****


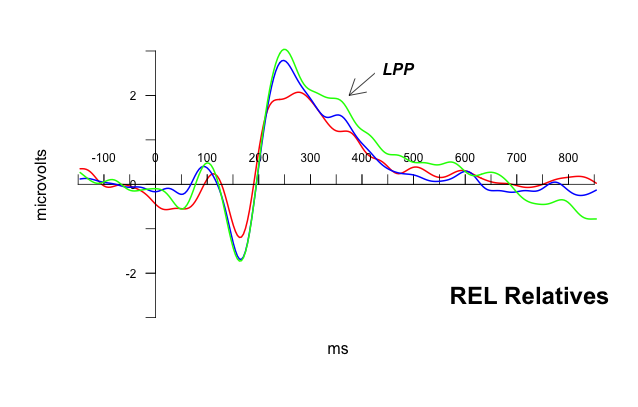


*Figure S2. Stimulus Order Effects on Encoding ERP Components*. P1 & N1 ERPs (left panel) and LPP ERPs (right panel) to first stimuli (red), second stimuli (blue), and third stimuli (green) for schizophrenia probands (PSZ), controls (CTRL), and relatives of PSZ (REL). P1 & N1 waveforms are derived from averages across electrodes PO4 and PO8; LPP waveforms are averages across electrodes FC1, C2, CP1. P1 responses to first stimuli were larger than those to second stimuli for PSZ alone. CTRL and REL showed presentation order-by-time window interaction effects, whereas PSZ did not. **p* < .05, ***p < .01, ****p*<.001.*

**PSZ**

**PSZ**

**CTRL**

**CTRL**

**REL**

**REL**


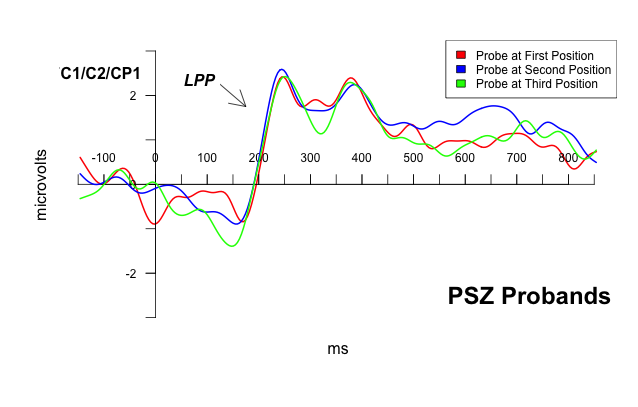

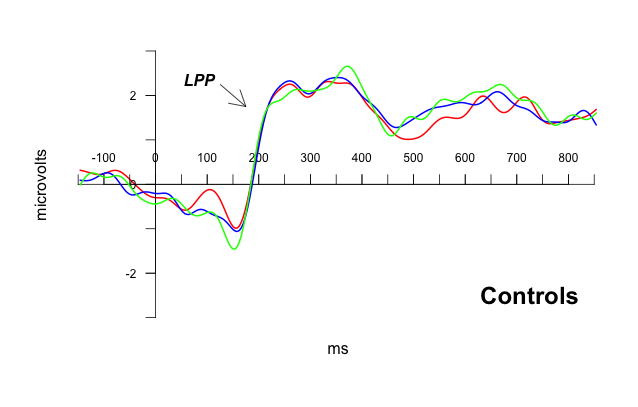

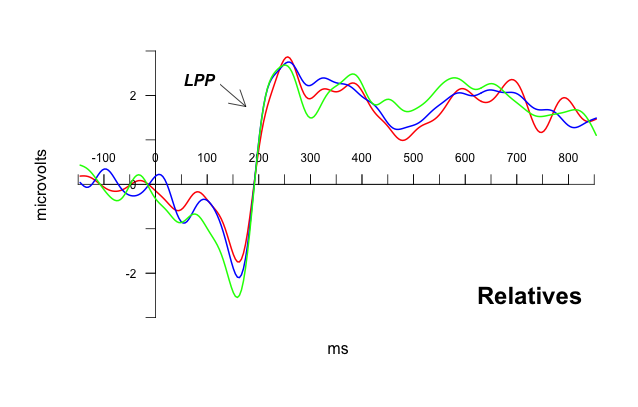


*Figure S3. Stimulus Order Effects on Retrieval LPP Component.* LPP ERPs to probes at previous 1^st^ stimulus location (red), 2^nd^ stimulus location (blue), and 3^rd^ stimulus location, (green) for schizophrenia probands (PSZ), controls (CTRL), and relatives of PSZ (REL). Waveforms are averages across electrodes FC1, C2, CP1. LPP responses showed a main effect of probe order for all groups combined, but neither main effects nor interaction effects for the individual groups.

**PSZ**

**CTRL**

**REL**
